# Mindful Emotion Regulation: Exploring the Neurocognitive Mechanisms behind Mindfulness

**DOI:** 10.1155/2015/670724

**Published:** 2015-06-07

**Authors:** Alessandro Grecucci, Edoardo Pappaianni, Roma Siugzdaite, Anthony Theuninck, Remo Job

**Affiliations:** ^1^Department of Cognitive Science (DiSCoF), University of Trento, Corso Bettini 84, Rovereto, 38068 Trentino, Italy; ^2^Department of Data Analysis, Faculty of Psychology and Pedagogical Sciences, Ghent University, 1 Henri Dunantlaan, 9000 Ghent, Belgium; ^3^Oxleas NHS Foundation Trust, Pinewood House, Pinewood Place, Dartford, Kent DA2 7WG, UK

## Abstract

The purpose of this paper is to review some of the psychological and neural mechanisms behind mindfulness practice in order to explore the unique factors that account for its positive impact on emotional regulation and health. After reviewing the mechanisms of mindfulness and its effects on clinical populations we will consider how the practice of mindfulness contributes to the regulation of emotions. We argue that mindfulness has achieved effective outcomes in the treatment of anxiety, depression, and other psychopathologies through the contribution of mindfulness to emotional regulation. We consider the unique factors that mindfulness meditation brings to the process of emotion regulation that may account for its effectiveness. We review experimental evidence that points towards the unique effects of mindfulness specifically operating over and above the regulatory effects of cognitive reappraisal mechanisms. A neuroanatomical circuit that leads to mindful emotion regulation is also suggested. This paper thereby aims to contribute to proposed models of mindfulness for research and theory building by proposing a specific model for the unique psychological and neural processes involved in mindful detachment that account for the effects of mindfulness over and above the effects accounted for by other well-established emotional regulation processes such as cognitive reappraisal.

## 1. Definition of Mindfulness

The practice of meditation has exploded in popularity around the world becoming one of the most widely used psychological techniques and disciplines [1]. In the last twenty years there has been a drastic increase in clinical interventions that take advantage of meditation skills, especially mindfulness meditation. According to Salmon et al. [2], more than 240 hospitals and international clinics offered mindfulness-based stress reduction (MBSR) and this number has increased since. MBSR was developed in clinics by Jon Kabat-Zinn at the University of Massachusetts Medical Center to facilitate adaptation to medical illness that provides systematic training in mindfulness as a self-regulation approach to stress reduction and emotion management [3]. Clinical applications of mindfulness have become widespread since the introduction of the mindfulness-based stress reduction (MBSR), a treatment program originally developed for the management of chronic pain [4–6]. MBSR has subsequently been applied to reduce the psychological morbidity in emotional and behavioral disorders and for the treatment of chronic mental illness [7], fibromyalgia [8], anxiety and panic attacks [9, 10], mood swings and stress in cancer patients [11], binge eating disorder [12], and multiple sclerosis [13].

Originally derived from Pali, the language of Buddhist psychology, the term “mindfulness” comes from the combination of two words, Sati which means “awareness” and Samprajanya, “clear comprehension,” thus indicating a way of being aware and attending to what happens. Mindfulness is considered to originate from eastern introspective spiritual practices, mainly Buddhism. In the most general sense it means to “pay attention with patience and care to what happens” [14, 15] and can be cultivated through meditation. However, you can find forms of mindfulness and meditation in almost all of the world's religions, such as yogic meditation in the Hindu tradition, kabbalah meditation in Judaism, contemplative prayer in Christianity, and Sufi meditation in Islam [16].

Adopted in western societies amongst enthusiasts of transcendental and metaphysical practices, mindfulness increasingly attracted scientific research to become recognized as an effective psychological practice for stress, pain management, and mental health problems [17].

Models of psychopathology recognize that automatic behavior without conscious attention to behavior (mindlessness) is associated with intrusive and ruminative thoughts about past or future events, leading to distressed states of mind, passivity, and the repetition of habitual coping patterns [18]. According to Kabat-Zinn, mindfulness meditation addresses this state of mind by cultivating a state of mind that is “on purpose” and “attentive” to reduce “mind wandering” (see [19] for the negative effects of mind wandering).

Mindfulness has been defined as a “nonelaborative, nonjudgmental, present-centered awareness in which each thought, feeling, or sensation that arises in the attentional field is acknowledged and accepted as it is” [20]. Kabat-Zinn [21] proposed a list of attitudes and behaviors that help develop being mindful in everyday life:

1. nonjudging: being an impartial witness to your own experiences without premature conclusions;
2. patience: letting things unfold in their own time;
3. beginner's mind: being receptive to new possibilities and not getting stuck in a rut of your own expertise;
4. trust: developing a basic trust in yourself and your feelings;
5. not striving: paying attention to how you are right now however that is;
6. accepting: seeing things as they actually are in the present;
7. letting go: letting go is a way of letting things be, of accepting things as they are.

Mindfulness-based stress reduction therapy is only one of many western adaptations of mindfulness practices applied in clinical settings. While mindfulness practice takes many forms, one common practice, especially in mindfulness-based therapies, is mindfulness of the breath. In a typical practice instruction, Kabat-Zinn suggests that participants sit comfortably with eyes closed and direct their attention to the physical sensations of breathing by simply noticing it, paying attention to it, and being aware of it. When thoughts, emotions, physical feelings, or external sounds occur, practitioners are instructed to accept them and allow the recognition of these stimuli to come and go without judging or getting involved. When attention has drifted off and is becoming caught up in thoughts or feelings it is advised that the practitioner notes this drift and gently brings attention back to breathing.

The defining psychological processes in mindfulness meditation are the unique attentional focus and attitudinal stance taken by the practitioner. The philosopher Husserl described the basic assumption of mindfulness as “paying attention to the experience as it presents itself without any interpretational filtering.”

This first component of mindfulness requires awareness and controlled effort to maintain full conscious focus in the “present moment” without wandering into thoughts of the past or the future. Mindfulness meditation seeks to cultivate this skill by first building awareness or “sustained attention” [22, 23], namely, the ability to maintain attentional focus on one object for a sustained period of time. Mindfulness traditionally focuses attention on the breath without repressing the flow of thoughts, feelings, and sensations that may supervene on the attentional focus. With repeated practice meditators report a capacity to focus and sustain their attention whilst experiencing a decreased frequency of distractions or intrusions into their attentional field during both formal meditation and everyday life situations [24].

## 2. Evidence for Psychological Mechanisms in Mindfulness

A considerable number of studies have empirically documented better performance during attentional tasks in meditators compared to controls [25–28].

The next process, closely related to the maintenance of attention, involves attentional switching, redirecting attentional drift back to the breath. Mindfulness meditation requires that this switching and refocusing is conducted with an attitude that involves nonjudgment and openness to current experience [29], an accepting state of mind that has been referred to as “intimate detachment” ([30] quoted in [31]) or “decentering” [32]. The meditator therefore does not engage with the content of the thoughts and feelings but aims to experience these as raw and unprocessed. In other words the attentional strategy implies a direct experience of events in the mind and body without being captured by them [33]. This observational stance has also been referred to as a metacognitive skill characterized by a decentering from environmental and internal psychophysiological stimuli or processes to produce a reflective space in which new ways of perceiving and responding become possible, rather than enacting habitual automatic or ruminative patterns [14]. In their systematic review Chiesa et al. [29] noted that neuropsychological testing supported these attentional processes in mindfulness meditation, although supportive findings were not observed in all studies, which the authors attributed to inconsistent operationalization and methodological limitations across studies.

One of the consequences of attentional focus in mindfulness meditation is the increase in body awareness by practitioners attending to internal perceptions, developing their understanding of subtle sensations and perceptions of their body [34, 35], with studies based on self-administered questionnaires showing a significant increase in body awareness in practiced meditators (see [36]).

Various neuroimaging studies support the above psychological observations and provide insight into the neurological processes activated by mindfulness meditation. We will consider these studies in the next section.

## 3. Neuroimaging Evidence for Mindfulness Mechanisms

Neuroimaging techniques have allowed scholars to uncover the neural mechanisms behind attentional and body awareness processes. Hölzel and colleagues [34] argued that mindfulness is underpinned by several distinct but interconnected neural mechanisms as hypothesized by Kabat-Zinn [18]. The dorsal medial prefrontal cortex (dmPFC) and the anterior cingulate cortex (ACC) in both hemispheres are activated in mindfulness meditation. The cingulate cortex plays a primary role in the fusion of attention, motivation, and motor control [37]. The rostral (ACC) part is activated in tasks with an emotional overload, whereas the dorsal (dmPFC) part is triggered by cognitive control tasks [38]. It is reasonable to assume that when a mental event such as a thought or a memory interferes with the attentional focus, the ACC may contribute to the maintenance of attention, alerting neural systems to solve the conflict by a top-down regulation mechanism that prioritizes cognitive control [39, 40]. According to Hölzel and colleagues [34], the area that shows more pronounced activation in experienced meditators while they are focusing their attention is precisely the rostral ACC, emphasizing the importance of this regulatory structure during meditation. Tang and colleagues [41] showed that five days of integrative body-mind training are sufficient for its effective activation during a resting state.

In a further study by Grant et al. [42] using structural MRI methods, they showed that cortical thickness in the dorsal ACC of experienced meditators was greater than that of control subjects. Furthermore, Tang and colleagues [41] have shown that eleven hours of integrative body-mind training (another form of mindfulness practice) is sufficient to observe improved integrity and efficiency of white matter in the corona radiata. Corona radiata is an important white-matter tract connecting the ACC to other structures, so its communication efficiency plays a key role in modulating brain activity in ACC. Thus IBMT can be a way to improve self-regulation and to reduce various mental disorders [43].

Another line of experiments confirmed that mindfulness increases activity and structural properties of brain regions connected to body awareness. For example, Farb and colleagues [44] found a greater activation of the insula (a region involved in proprioception and viscerosomatic processing) in individuals trained in mindfulness-based stress reduction, as compared to the control group. In addition, it was discovered that the thickness of the insula is greater in experienced meditators as compared to controls [45] and that there is a greater concentration of gray matter in the right anterior insula [46].

Taken together, these studies provide evidence that mindfulness practice affects psychological and neural processes and systems that improve attentional capacity and body awareness and engage cognitive control processes.

## 4. Clinical Evidence for the Effects of Mindfulness

According to Bishop [3], randomized clinical trials confirmed the positive effect of meditation and provide encouraging results with patients showing a significant reduction in psychological morbidity associated with mental illness [47–49] and a decrease in stress level, resulting in increased emotional well-being.

Several psychological treatments of different schools have explicitly or implicitly incorporated mindfulness principles. Among these are

1. dialectical behavior therapy (DBT) [50], an approach that develops emotion-regulation skills across a variety of mental disorders including borderline personality disorder by incorporating mindfulness exercises and attentiveness to emotions, thoughts, and feelings in addition to cognitive and behavioral treatment tasks [51];
2. mindfulness-based cognitive therapy (MBCT) [52], which combines a mindfulness training program with cognitive restructuring tasks to reduce relapses in depression [53];
3. acceptance and commitment therapy (ACT) [54], which helps patients modify their relationships with inner experience through taking an accepting observing position of thoughts, feelings, and sensations and take committed action in accordance with their own values, similar to a mindful attitude;
4. intensive short-term dynamic psychotherapy (ISTDP) [55, 56], which guides the patient in a moment-by-moment focusing to develop the patient's capacity to observe and attend to emotional and bodily responses within the therapeutic and other relationships;
5. relational and attachment focused psychoanalysis [57, 58], in which the therapist mindfully distances from his/her own responses in order to reflect on countertransference, guiding interpretations, and responses to the patient, thereby inviting the patient into a similar mindful stance toward themselves, the therapist, and others.

After a decade of observations and studies, therapeutic mindfulness approaches have shown positive effects in patients with anxiety disorders [59, 60], posttraumatic stress disorder [61], substance abuse [62, 63], eating disorders [12, 64], depression [53], and personality disorders [41].

Neuroimaging studies further support the therapeutic effects of applying mindfulness in the treatment of clinical populations. In patients with social anxiety disorder, Goldin and colleagues [65] found improvements in attentional skills indicated by increased activation in the zone modulated by attention in the parietal regions, with a concomitant decrease in negative emotions.

In further research Goldin and colleagues [65] found that a course of MBCT in patients with a history of suicidal depression supported stable neural functioning in relative left-frontal activation indicating the maintenance of their level of positive affective style compared to patients receiving treatment as usual who showed decreased activation and thus worsening positive affectivity.

Despite the evidence of the effectiveness of mindfulness in psychopathological disorders, the precise and unique neurocognitive mechanism by which these effects occur requires study and theoretical explication. To support the development of therapeutic methods it is necessary to distinguish the unique mechanisms contributed by mindfulness over and above common therapeutic factors. Clarity about unique factors will allow precise mindfulness techniques to be introduced into a variety of treatment programs and settings, therefore relying less on applying wholesale mindfulness treatment packages in order for patients to benefit from its unique factors. Whilst some therapeutic methods focus on mindfulness meditation as a specific practice within the treatment such as MBSR, MBCT, and DBT, other therapies include mindfulness attitudes and techniques as integrated within the therapeutic discourse such as ACT, ISTDP, and relational and attachment focused psychoanalysis. The delivery of mindfulness techniques may therefore vary but knowing what specific active factor mindfulness delivers would aid in further focusing the use of mindfulness in these treatments in order to assure efficiency and effectiveness in treatment delivery.

## 5. Models and Hypotheses about the Mechanisms of Mindfulness Meditation

Despite the amount of evidence on the psychological and neural effects of mindfulness, research is required on the specific neurocognitive mechanisms that underlie this meditative practice and what unique mechanisms may account for its specific therapeutic effects over and above the therapeutic factors that are common to psychological therapies generally [17].

To investigate the active factors in mindfulness meditation, it is necessary for research to be guided by theoretical models of mindfulness in order to refine understanding about unique and common therapeutic factors.

The evidence reviewed thus far points to mindfulness being characterized by an increased attention to both external (environmental) and internal (body awareness) stimuli. Mindfulness meditation promotes an adaptive observational stance toward inner experience and responses to stimuli characterized by a compassionate or calm stance where the aim is not to give in to, avoid, or control the experience or stimuli but to maintain observational exposure from a decentered position [20, 66, 67]. Mindfulness therefore aims to create psychological distance between the observing self and the emotion to enable emotional regulation that minimizes negative consequences [5]. But what are the processes that occur during the observing stance that support emotional regulation?

Shapiro and colleagues [14] proposed that mindfulness comprises three interacting processes of intention, attention, and attitude. The meditator is guided by an* intention* to self-regulate, to self-explore, and finally to self-liberate using an* attentive* moment-by-moment focus on the contents of consciousness with an* attitude* of compassionate open-heartedness. The authors assert that this is a developmental process through which a person increasingly develops an objective observing position of self and others through which metacognitive insights are obtained about “I am not my pain” and “my sense of self is ever-changing.” This model implies the use of cognitive reappraisal as a key means to regulate emotional states, namely, that the practitioner is “reperceiving” or reappraising their relationship with their emotional experience or habitual responses and sense of self so that the person is no longer fused or identified with their emotion or state but reframes it as an impermanent mental phenomenon.

In the model proposed by Hölzel and colleagues [34] sustained attention and body awareness are proposed as processes by which the meditator identifies and is exposed to habitual reactions and previously avoided or suppressed internal and external experiences. Habitual responses are prevented by taking a nonreactive stance that is focused on experiencing thereby allowing for extinction of the habitual response. The passing of habitual responses brings to mind the transitory nature of experiencing perceptions, emotions, and cognitions and allows for a change in perspective of the self. Emotional regulation is thus achieved by a mindful exposure and response prevention requiring experiencing and cognitive reappraisal of aversive stimuli as transitory or even as positive or meaningful.

Both of these models outline both cognitive and experiential processes that enable emotional regulation through mindfulness meditation. Cognitive processes are involved in purposeful focusing of attention and reappraisals that allow for a new way of understanding the person's observing position and the transitory nature of external and internal experience. These may be referred to as top-down processes whereby the meditator engages executive attention and control in order to engage with experience. In addition to this the models refer to bottom-up experiential processes whereby the person remains conscious of raw unprocessed present-centered experiencing that prevents or interrupts habitual responses and is enabled by an attitude of nonjudgment, compassion, and open curiosity. In order to enable the experiential process, cognitive reappraisal may be required of self and the situation in order to take on the appropriate attitude that will allow experiencing.

These theoretical models of mindfulness allow us to begin to ask questions about the relative contribution of different mechanisms in producing the emotional regulatory effects of mindfulness meditation. It is proposed that the different facets of mindfulness meditation (intention, attention, attitude, body awareness, reappraisal, and changes in perspectives on self) employ both cognitive and experiential mechanisms that enable internal experience of self and the situation to be regulated. Whilst mindfulness meditation methods are unique (e.g., focusing on observing the breath) the question raised is whether the mechanisms of change are unique and if so, which mechanisms are specifically unique and effective in producing the mindfulness effects? Clarity about the mechanisms of change is relevant to the application of mindfulness techniques to other therapies.

Does mindfulness regulate our emotions primarily because it changes our way of thinking about self and the situation, namely, via cognitive reappraisal of an emotional situation as positive [34, 68], or because it produces an intimately detached or decentered accepting point of view that allows for raw experiencing (see, e.g., [20, 69])? Modinos and colleagues [70] in a study of neuroimaging have shown that the dispositional traits of mindfulness are positively correlated with the activation of the dorsomedial prefrontal cortex (dmPFC) when reappraisal was used. However other studies have shown opposite results, that is, a substantially lower activity in dmPFC during reappraisal in mindfulness meditators [71, 72]. One can ask then, if mindfulness is able to regulate our emotions, what are the mechanisms by which this happens? Does mindfulness produce emotion regulation through cognitive reappraisal alone or through other regulatory mechanisms as well? In other words, does mindfulness have an effect primarily using a cognitive mechanism or an experiential mechanism? We turn our attention now towards experimental evidence and the light this may shed on the mechanisms of mindfulness to produce emotion regulation.

In recent years, for its widespread application in everyone's life, the processes by which we regulate our emotions have caught the attention of many researchers. We daily regulate our emotions in order to channel them into adaptive actions. According to Gross, emotional regulation is nothing more than a set of processes through which we regulate our emotions [73]. The emotional regulation process either mitigates, enhances, or simply maintains stability of an emotion. People are able to adjust both negative and positive emotions by attenuating or increasing them.

## 6. Experimental Evidence of a Mindful Emotional Regulation: Exploring the Unique Psychological and Neural Processes Involved in Mindfulness

In the last two decades clinicians have shown that mindfulness training can improve emotional disorders [74] and negative mood [75] and reduce physiological responses such as skin conductance [76] and amygdala activity [67]. These findings point to the emotional regulatory function of mindfulness for which recent studies have provided direct experimental evidence.

Early investigations [76] into the effects of mindfulness meditation recorded galvanic skin response of a group of experienced meditators and of controls while viewing unpleasant video. Results showed that, beside a first rise in galvanic skin response similar in both groups, meditators were able to lower their arousal to a greater degree than controls. More recent investigations by Taylor and colleagues [77] similarly tested the skills of mindfulness meditators to reduce the emotional intensity they felt in response to an unpleasant image. The authors tested twelve long-time meditators and ten beginner meditators as they looked at pleasant, unpleasant, and neutral images, recording the task with fMRI. The results showed that both groups were able to experience reduced emotional reactions on a subjective and neural level using mindfulness whilst looking at the images. Yet Taylor and colleagues [77] further observed two different emotional regulation mechanisms that depend on the degree of experience of the meditator. In experienced meditators the medial prefrontal and posterior cingulate cortices were deactivated and did not influence brain regions involved in emotional reactivity during emotional processing. However, for beginner meditators, mindfulness induced a downregulation of the left amygdala during emotional processing. Brefczynski-Lewis and colleagues' [78] study with sound stimuli similarly found a negative correlation between the length of meditation hours and activation of the right amygdala while listening to unpleasant stimuli. These studies therefore suggest that practiced mindfulness skills lead to emotional regulation through accepting emotional states and enhancing present-moment awareness, whilst beginner mindfulness skills appear to rely on higher cortical brain regions to control low-level affective cerebral systems. The mechanism of self-regulation utilized during mindfulness therefore depends on the degree to which the meditator has practiced mindfulness.

In examinations of individuals with higher dispositional mindfulness Modinos and colleagues [70] showed that greater dorsomedial prefrontal cortex (dmPFC) activation occurred during a task of reappraising negative stimuli compared to activation during a task of merely attending to the negative stimuli. This dmPFC activation was inversely correlated with the amygdala response to negative scenes thus offering brain image evidence for the role of cognitive control strategies during mindfulness in regulating emotional arousal in people with higher dispositional mindfulness.

Therefore research suggests that beginner mindfulness skills as well as high dispositional mindfulness traits are more likely to involve top-down emotional regulation mechanisms based on cognitive control mechanisms, whilst experienced meditators are more likely to use bottom-up regulation mechanisms that rely on the perceptual system rather than cognitive system. Mindfulness is a skill and the emotional regulatory mechanism it deploys therefore appears to depend on the degree of meditation practice rather than purely on the application of the mindfulness method.

Cognitive reappraisal has been suggested as a core cognitive control skill whereby mindfulness practice may regulate emotions [34]. However such skill is not unique to mindfulness meditation and is a core feature taught to patients in numerous psychological therapies. To explore the emotional regulatory mechanism unique and therefore specific to mindfulness, recent experimental investigations have examined the relative contribution of cognitive reappraisal mechanisms versus the mindful state of intimate detachment in achieving emotional regulation during mindfulness meditation.

Opialla and colleagues [79] recently directly compared novice subjects using mindfulness versus subjects using only cognitive reappraisal whilst viewing negative emotional pictures. The researchers observed the medial prefrontal cortex activated in both groups indicating that similar top-down neural mechanisms for emotional regulation occur in the mindfulness process as in the cognitive reappraisal process.

However, Opialla and colleagues [79] further observed that the mindfulness group showed selective insula activation, a region shown to be involved in the regulation of the experience of emotions [80–82], whereas the caudate was selectively active in the cognitive reappraisal group, which is a region usually associated with cognitive control. These results therefore suggest that even in a group of novice mindfulness meditators, emotion-focused or experiential mechanisms of emotional regulation operate which are not present in subjects who purely employ cognitive reappraisal and for whom cognitive control mechanisms are more active.

Research is therefore pointing more towards mindfulness practice contributing a unique bottom-up experiential process in regulating emotions that employs different pathways to that utilized by cognitive reappraisal. Grecucci and colleagues [69] further explored the differential contribution of cognitive reappraisal and mindful detachment when accounting for the frequency of practice. They compared emotional experience and behavior in response to socially unpleasant stimuli in both practiced meditators and beginner meditators. Both groups' responses were measured when they used alternately cognitive reappraisal to cope with an experimental situation of social unfairness and when they used intimate detachment (the unique mindfulness experiential strategy) when dealing with a similar social unfairness. Whilst both groups were able to regulate their emotionally driven behaviors, experienced meditators were more able to regulate themselves than beginners when using the experiential “intimate detachment” strategy, whilst neither group outperformed the other in emotion regulation when using a cognitive reappraisal strategy. The evidence suggests that greater practice in mindfulness therefore does not necessarily enhance a person's capacity to self-regulate using cognitive reappraisal but does enhance the use of intimate detachment as a means of self-regulation.

These findings substantiate the growing evidence that mindfulness meditation enables emotional and physiological regulation. Furthermore the findings suggest that one of the unique components of mindfulness practice is to enable emotional regulation through the practice of intimate detachment that relies on bottom-up neural activations and pathways. The effectiveness of intimate detachment is also sensitive to the practice effect with more practiced meditators being more able to regulate their emotions and responses. Yet what specific mechanism may be proposed that could account for the neural and psychological process of intimate detachment?

In line with the above cited and other recent evidence [81, 83–85], we outline a neural circuit comprising the prefrontal cortex (PFC), the anterior cingulate cortex (ACC), the amygdala (A), and the insula (I) that are involved in the unique processes of mindful emotion regulation. This circuit includes “top-down” control regions such as the PFC and the ACC and more “bottom-up” emotional regions such as the I and the A.

These two neural networks (PFC-ACC and A-I) interact via connective neural structures such as the corona radiata. Focused, sustained, intimately detached attention on emotional states (activation of PFC and ACC) leads to increased awareness and knowledge of emotions (I and A activation). The mindfulness practitioner's effort to stop the generation of thought and to pay attention to internal perceptions and emotions is reflected in PFC and ACC activation. Ruminative thoughts triggered by unpleasant emotions further reinforce those negative emotions leading to a maintenance or escalation of negative emotions. Breaking this cycle by redirecting intimately detached attention to emotions stops this vicious cycle. We propose that activation of PFC impulse control and ACC stimulus discrimination and attentional focus are some of the neuropsychological processes that enable intimate detachment. Yet, with increased frequency of mindfulness practice there is greater activation of the ACC [34, 41, 42] and less utilisation of PFC functions, which signifies a greater reliance on perceptual stimulus discrimination rather than active cognitive control strategies. The PFC and ACC regions are activated in the service of attuning to emotions through greater regulatory connectivity with the A-I regions. Whilst the PFC region can contribute to emotional regulation via cognitive reappraisal, this is not unique to mindfulness. Instead, it is the increasing activation of the ACC functions of stimulus discrimination, attention to emotional stimuli, and resolution of stimulus conflict that are more involved in the unique process of intimate detachment that enables emotional regulation. This particular utilisation of the PFC-ACC regions and its connectivity with the I-A regions is also enhanced by practice.

This broad neurological model for the effects and mechanism of intimate detachment in mindfulness offers a structure for further research and model building. It may guide future research enquiry about more detailed neuropsychological processes (such as different attentional processes) that are required in order to enable the state of intimate detachment. For example, are certain attentional skills (attentional focusing versus shifting skills) more or less important in developing the capacity for intimate detachment? What are the specific psychological components characterised by greater ACC activation in practiced meditators? How can this enhance our understanding of how patients could achieve the experiential emotional regulatory state of intimate detachment or decentering?

## 7. Conclusions

Mindfulness meditation has become a widespread, accessible, and effective method for improving mental and physical well-being. In this paper we reviewed the psychological and neural mechanisms believed to be at the heart of this method. Current evidence for the components of mindfulness meditation refers to attentional processes, body awareness, and cognitive regulatory processes such as cognitive reappraisal. Clinical effectiveness across a range of psychological disorders, stress, and pain conditions has been evidenced but without specific understanding of how mindfulness meditation enables such clinical recovery. More recently models of mindfulness meditation have been put forward in order to support research enquiry into the specific and unique components that characterize it. Such models allow for more rigorous examination and operationalization of the method to guide research. Increasingly investigators are focusing on the impact mindfulness has on emotional regulation which accounts for the effects on mental health. Two component processes that have attracted examination are emotional regulation through the process of cognitive appraisal and through the process of intimate detachment, the latter being the more experiential aspect of mindfulness meditation. Whilst cognitive reappraisal plays a significant role in mindfulness, we have highlighted evidence that shows that it is the stance of intimate distancing that is unique to mindfulness in enabling emotional regulation and that it is this unique method that is especially enhanced in experienced meditators. Based on the evidence we have proposed a neural circuit whereby the stance of intimate detachment operates. This model may enable researchers to focus future investigations on neuropsychological processes that contribute to developing the state and skill of intimate detachment. Further refinement of our understanding of how intimate detachment is achieved may also aid the application of this unique aspect of mindfulness methods within various psychological therapies.
